# The Impact of Iron Overload and Ferroptosis on Reproductive Disorders in Humans: Implications for Preeclampsia

**DOI:** 10.3390/ijms20133283

**Published:** 2019-07-04

**Authors:** Shu-Wing Ng, Sam G. Norwitz, Errol R. Norwitz

**Affiliations:** 1 Department of Obstetrics & Gynecology, Tufts University School of Medicine, Boston, MA 02111, USA; 2 Mother Infant Research Institute, Tufts Medical Center, Boston, MA 02111, USA; 3 Washington University, St. Louis, MO 63130, USA

**Keywords:** ferroptosis, hypoxia/reperfusion injury, maternal-fetal interface, preeclampsia

## Abstract

Iron is an essential element for the survival of most organisms, including humans. Demand for iron increases significantly during pregnancy to support growth and development of the fetus. Paradoxically, epidemiologic studies have shown that excessive iron intake and/or high iron status can be detrimental to pregnancy and is associated with reproductive disorders ranging from endometriosis to preeclampsia. Reproductive complications resulting from iron deficiency have been reviewed elsewhere. Here, we focus on reproductive disorders associated with iron overload and the contribution of ferroptosis—programmed cell death mediated by iron-dependent lipid peroxidation within cell membranes—using preeclampsia as a model system. We propose that the clinical expressions of many reproductive disorders and pregnancy complications may be due to an underlying ferroptopathy (elemental iron-associated disease), characterized by a dysregulation in iron homeostasis leading to excessive ferroptosis.

## 1. Introduction

Iron is the most common transition metal in living cells. An average person requires 20 mg of elemental iron each day just to make enough erythrocytes (red blood cells) [1]. In addition to erythropoiesis, iron is also essential for biological processes within all cells, including energy production and DNA replication and repair. Because of its propensity to reversibly gain or lose single electrons by transitioning between different oxidation states, iron serves as an essential element for a wide variety of vital enzymatic redox reactions within cells.

Average daily iron absorption in premenopausal women is about twice that of men, due mostly to blood loss during menstruation and episodes of increased demand during pregnancy and lactation [2]. The average daily iron requirement in pregnancy increases to around 1000–1200 mg to meet the maternal and feto-placental need for erythropoiesis, growth, and development [3]. This is a large amount of iron, especially when compared with the average total body iron content of 2200 mg and the 1.3 mg of iron absorbed per day by nonpregnant women. Iron-deficiency anemia is the most common nutritional deficiency worldwide, and is associated with pregnancy complications such as low birthweight (LBW) and preterm birth (PTB), as well as impaired neurodevelopment and immune function in infants, children, and adolescents [4,5]. For these reasons, much attention has focused on preventing iron-deficiency anemia, and iron supplementation has become a routine recommendation for all women throughout pregnancy. Recent studies have raised questions about the value of iron supplementation in women who are iron-replete and non-anemic and suggested that excess iron intake may paradoxically increase the risk of reproductive disorders [3,6].

In this review, we summarize the current understanding of the factors regulating systemic and cellular iron trafficking and how these change under conditions of health, pregnancy, and disease. We discuss the reproductive risks associated with excessive iron intake and/or high iron status. Lastly, we present a hypothesis that may explain the underlying molecular basis for excess iron-mediated disorders of women’s health and reproduction, using preeclampsia (PE) as a model system. In brief, we hypothesize that such reproductive disorders represent an underlying ferroptopathy characterized by a dysregulation in iron homeostasis leading to excessive ferroptosis, a recently described process of programmed cell death mediated by iron-dependent lipid peroxidation of cell membranes.

## 2. Systemic and Intracellular Iron Homeostasis

### 2.1. Regulation of Systemic Iron Homeostasis

**Figure 1:**
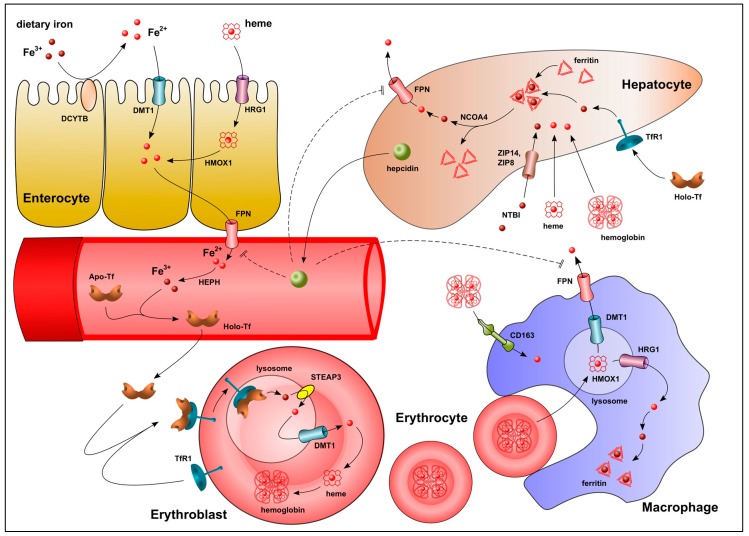
Schematic representation of the factors involved in systemic iron absorption, utilization, storage, and recycling.

The factors regulating iron absorption, utilization, storage, and recycling are summarized in Figure 1. Duodenal enterocytes take up dietary iron, of which 85–90% is absorbed as non-heme iron [7]. The major iron uptake transporter is DMT1 (divalent metal transporter 1 [SLC11A2]), which is located on the apical aspect of intestinal cells. Tissue-specific deletion of the *Dmt1* gene in mice results in a 90% impairment of iron absorption leading to severe iron-deficiency anemia [8,9]. A ferrireductase enzyme, such as duodenal cytochrome B (DCYTB), located at the apical membrane of enterocytes is required to reduce ferric iron (Fe^3+^) in food to ferrous iron (Fe^2+^) to enable DMT1 transport. The importance of DCYTB in this process is supported by the observation that *Dcytb* mRNA expression is markedly upregulated in the intestines of iron-deficient mice [10]. Less is known about the absorption of heme iron in the human small intestine, although studies suggest that it is mediated by HRG1 (heme-responsive gene-1) receptor-mediated endocytosis [11] and the subsequent release of iron within enterocytes by the action of HMOX1 (heme oxygenase 1) [12]. Ferroportin (FPN [SLC40A1]), the only known human iron exporter, then transports iron out of enterocytes into the blood [13,14,15]. Intestine-specific deletion of the *Fpn* gene in mice results in an accumulation of iron within duodenal enterocytes and, subsequently, in severe anemia, demonstrating the importance of FPN in intestinal iron export [16]. A ferroxidase enzyme, such as HEPH (hephaestin), then oxidizes Fe^2+^ to Fe^3+^ [17], which can bind to the transporter protein transferrin (Tf [Apo-Tf]) and be transported as Tf-Fe^3+^ (Holo-Tf) through the blood to target organs.

The majority of circulating iron carried by Tf is delivered to erythroblast progenitor cells in the bone marrow for differentiation into erythrocytes. TfR1 (transferrin receptor 1), located on the surface of erythroblasts, recognizes the iron-loaded holo-Tf and the complex is internalized into endosomes. The acidic environment within the endosomes causes the Fe^3+^ to dissociate from Tf. It is then reduced to Fe^2+^ by the endosomal ferrireductase, STEAP3 (six-transmembrane epithelial antigen of prostate 3), and transported out of the endosomes and into the cytosol by DMT1 [18,19]. In erythroblasts, almost all of the iron that enters the cytosol is directed to mitochondria for heme biosynthesis. There is also a “kiss and run” mechanism in which endosomal iron bypasses the cytosol and is transferred directly from DMT1 into the mitochondria [20]. Mitoferrin 1 (in developing erythroblasts) and mitoferrin 2 (in non-erythroid cells) are transport proteins located on the mitochondrial inner membranes that are responsible for taking up the iron [21]. They form a complex with FECH (ferrochelatase) and ABCB10 (ATP binding cassette super-family B member 10), which together insert iron into PPIX (protoporphyrin IX) to produce heme [22,23]. Heme is then exported out of mitochondria via isoform b of the FLVCR1 (feline leukemia virus subgroup C receptor-related protein 1) transport protein for incorporation into hemoglobin and other hemoproteins [24]. In vitro studies have shown that suppression of *Flvcr1b* gene expression results in mitochondrial heme accumulation and termination of erythroblast differentiation [25]. *Flvcr1* gene null mice die in utero due to a block in erythroblast differentiation, whereas embryos lacking only the 1a plasma membrane isoform have normal erythropoiesis [25,26].

Macrophages of the liver, spleen, and bone marrow—collectively known as the reticuloendothelial system (RES)—are primarily responsible for clearing and recycling iron from senescent or damaged erythrocytes [27]. Indeed, RES macrophages recycle about 25 mg of iron from erythrocytes each day, compared with only 1–2 mg of dietary iron absorbed by intestinal enterocytes [28]. Macrophages degrade erythrocytes via phagocytosis. Once in macrophage endosomes, heme is translocated to the cytosol via HRG1 [29] and broken down by HMOX1 to release its iron content [30]. HMOX1-mediated heme degradation also protects macrophages from cytotoxic heme, as *Hmox1* gene-deficient macrophages cannot survive erythrophagocytosis in vitro and *Hmox1* gene null mice have a complete loss of macrophages in their liver and spleen [31]. Macrophages can also scavenge hemoglobin released from intravascular hemolysis via CD163, a receptor expressed exclusively on the surface of monocytes and macrophages [32], and can scavenge heme in the circulation if bound to the glycoprotein hemopexin [33]. The scavenged heme is then catabolized by HMOX1 and the iron transported out of the cell via FPN.

While macrophages are responsible for the recycling of iron from erythrocytes, hemoglobin, and heme, the liver is the major storage organ for excess iron in the form of cytosolic heteropolymers, each of which is made up of 24 subunits of heavy (FTH1) and light (FTL) ferritin chains [34]. Hepatocytes take up all forms of iron, including Tf-bound iron (via TfR1), non-transferrin-bound iron (NTBI) [35] (via the transmembrane transporter proteins ZIP8 (Zrt- and Irt-like protein 8 [SLC39A8]) and ZIP14 (SLC39A14) [36,37]), hemoglobin, and heme. These are stored intracellularly bound to ferritin. In iron-deficient states, the iron stored in ferritin is mobilized by the interaction of NCOA4 (nuclear receptor coactivator 4) with FTH1. The complex is then degraded in autolysosomes, a process called ferritinophagy [38,39]. Besides carrying out the function of iron storage, hepatocytes also release iron via FPN together with CP (ceruloplasmin), a circulating ferroxidase enzyme [40]. Mice with hepatocyte-specific deletion of the *Fpn* gene show impaired hepatic iron mobilization and iron-deficiency anemia in response to phlebotomy or an iron-deficient diet [41]. Hepatocytes also regulate the iron homeostasis by producing the endocrine hormone hepcidin [42,43], which binds to FPN on the surface of macrophages, enterocytes, and hepatocytes, and initiates the internalization and degradation of FPN [44]. The hepcidin-FPN cycle is an important mechanism regulating systemic iron homeostasis. Hepcidin expression is upregulated in response to increased plasma iron, iron stores, and inflammation, and suppressed in the setting of anemia, the latter resulting in increased FPN protein levels in intestinal enterocytes to promote iron release into the blood. Some cases of hereditary hemochromatosis are caused by mutations in the *Fpn* gene, which impair the ability of hepcidin to bind FPN [45], resulting in increased saturation of Tf in the circulation, hepatic iron overload [46], and exocrine pancreatic failure [47]. *Fpn* gene expression is also regulated by a number of hepcidin-independent mechanisms, such as heme release from digested erythrocytes within macrophages [48], hypoxia, iron deficiency (mediated via HIF-2 (hypoxia-inducible factor 2) within duodenal enterocytes [49]), and inflammatory signals within the liver and spleen [50].

### 2.2. Regulation of Intracellular Iron Trafficking

**Figure 2:**
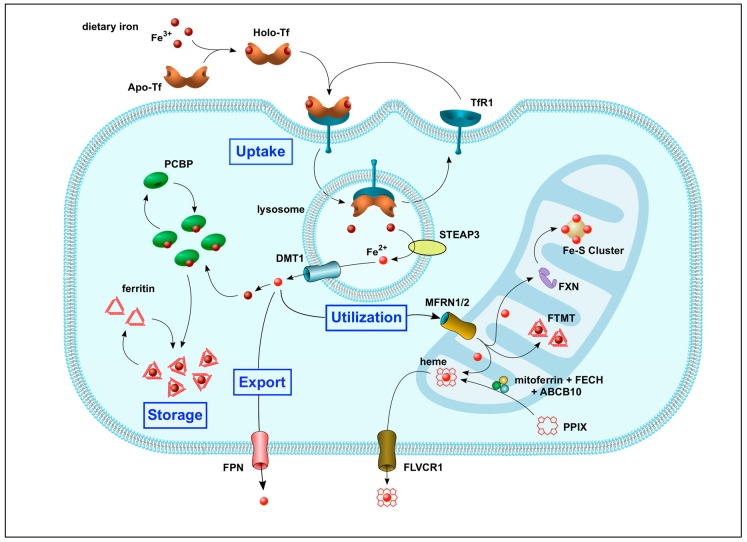
Schematic diagram showing iron import, export, utilization, and storage within cells.

Figure 2 illustrates iron trafficking within cells. Under normal physiological condition, cells primarily take up Tf-bound iron. In brief, holo-Tf binds to the high-affinity TfR1 on the cell surface, the complex is internalized by receptor-mediated endocytosis, the enzyme STEAP3 reduces Fe^3+^ to Fe^2+^, and the Fe^2+^ is exported into the cytosol by DMT1. Under conditions of iron overload, cells also take up iron in the form of NTBI via the ZIP8 and ZIP14 transporter proteins [36,51]. Once inside the cell, iron binds to chaperone proteins, such as the PCBPs (poly-(rC)-binding proteins), to prevent iron-mediated cytotoxicity, and thus becomes a part of the chelatable and redox-active intracellular labile iron pool [51,52].

In non-erythroid cells, 70–80% of cytosolic iron is incorporated into ferritin for storage [53]. Iron-loaded PCBP1 and PCBP2 form a stable ternary complex with ferritin during the iron transfer. This same mechanism is used to deliver iron to non-heme iron-requiring enzymes such as HIF1α (hypoxia-inducible factor 1α) [54]. When iron stores need to be mobilized, ferritin is bound to NCOA4 and transported to lysosomes for iron release via the aforementioned mechanism of ferritinophagy [39]. Excess iron is exported out of the cell via FPN [14].

Most of the iron in the cytosolic labile iron pool is transported into the mitochondrial matrix by MFRN1 (mitoferrin 1) or MFRN2 (mitoferrin 2), and is either stored as FTMT (mitoferritin) or incorporated into heme and iron-sulfur (Fe-S) clusters, which are required for energy production via oxidative phosphorylation [55]. Indeed, all four of the electron transport chain complexes in mitochondria require Fe-S clusters and heme moieties to function. In addition, Fe-S clusters and heme serve as cofactors for a variety of biological processes, including redox reactions, ribosome assembly, DNA damage repair, telomere maintenance, environmental sensing, and DNA replication [55]. Interestingly, the iron-chaperone protein FXN (frataxin), which is required for the assembly of Fe-S clusters, can also influence heme synthesis [56]. It has been suggested that FXN functions as a metabolic switch between these two key biosynthetic pathways, depending on which pathway is needed more urgently in the cell [57]. Furthermore, disruption of Fe-S cluster biosynthesis by *Fxn* gene knockout in mice unmasks a compensatory feedback mechanism to increase cellular and mitochondrial iron uptake (by upregulating *Tfr1* and *Mfrn2* gene expression) and decrease both cytosolic iron storage (by suppressing *Fth1* and *Ftl* gene expression) and export (by downregulating *Fpn* gene expression). This leads, in turn, to mitochondrial iron overload and an increase in oxidative stress—the damage done to cells when they are exposed to electronically unstable molecules called free radicals—at the expense of cytosolic iron deficiency [58]. In the same way, disruption of heme biosynthesis has also been shown to alter the expression of genes that favor iron uptake and mitochondrial iron accumulation [59]. Importantly, examples of gene mutations that disrupt mitochondrial iron homeostasis, leading to iron overload and a significant clinical phenotype, have been identified in humans [60,61,62], thus demonstrating the importance of this regulatory mechanism between the cytosol and mitochondria. Taken together, these data show that mitochondria play a vital role in cellular metabolism and that dysregulation of mitochondrial iron metabolism can have significant clinical impact [63], which is discussed in further detail in Section 4.3. below.

Similar to the hepcidin-FPN feedback loop that regulates systemic iron homeostasis, there exists an accompanying intracellular posttranscriptional mechanism, which is mediated by two iron regulatory proteins (IRP1 and IRP2). These IRPs bind to iron-responsive elements (IREs) within mRNAs encoding key iron regulatory proteins and allow for rapid modulation of their expression in response to intracellular iron levels [64]. The IRPs are not active when iron is abundant. However, when iron is deficient, IRPs bind to the IREs located in the 5′ untranslated region of the *Fth1*, *Ftl*, *Hif2α* and *Fpn* mRNAs to inhibit their translation. In contrast, binding of these same IRPs to the IREs located in the 3′ untranslated region of the *Tfr1* and *Dmt1* mRNAs serves to prevent their degradation, resulting in increased iron uptake and decreased iron storage and export. When iron is in excess, the IRE-binding activity of IRP1 is blocked by Fe-S clusters [65], whereas excess iron will cause IRP2 to bind to the iron and oxygen sensor, FBXL5 (F-Box and leucine-rich repeat protein 5), leading to its ubiquitination and proteasome degradation [66]. Double deletions of *Irp1* and *Irp2* are embryonic lethal [67]. Although the two IRP proteins show some functional redundancy, they have unique in vitro binding specificities. IRP1 is responsive to mitochondrial iron concentrations, while IRP2 is sensitive to cytoplasmic iron. There is also an interplay between oxygen tension and iron homeostasis within cells. IRP1 is functionally more active at high oxygen concentrations and subdued by low oxygen concentrations, an environment that favors assembly of Fe-S clusters and inhibition of IRP1 [68]. Hence, HIF2α, which is regulated predominantly by IRP1, promotes *Dmt1*, *Dcytb*, and *Fpn* gene transcription to maintain iron homeostasis under conditions of hypoxia and iron deficiency. In contrast, IRP2 is more active at low oxygen tension [69]. Under conditions of high oxygen tension and excess iron, IRP2 undergoes FBXL5-mediated ubiquitination and proteasomal degradation. Similarly, HIF1α and HIF2α proteins are also hydroxylated by iron-dependent prolyl hydroxylases and polyubiquitinated for proteasomal degradation when oxygen levels are high [70]. Thus, these two IRPs with different modes of activation regulate intracellular iron homeostasis over a wide range of oxygen concentrations.

## 3. Impact of Dysregulated Iron Homeostasis on Reproductive Outcomes

### 3.1. Elevated Maternal Serum Iron/Hemoglobin Levels Are Associated with Adverse Pregnancy Outcomes

The World Health Organization (WHO) estimates that globally over 40% of pregnant women are anemic and that more than half of these cases are due to iron deficiency [71]. As such, WHO recommends routine iron supplementation of 30–60 mg per day throughout pregnancy [72]. Recent studies have shown that multiple physiological adaptation occur in the mother during pregnancy to meet the higher demand for iron. Circulating hepcidin levels decrease and are nearly undetectable in the latter half of pregnancy, which promotes dietary iron absorption and mobilization of iron from tissue stores. These adaptations, along with the reduced loss of iron due to the cessation of menstruation during pregnancy, may actually limit the ability of feedback mechanisms to respond to environmental iron exposure and may enhance the vulnerability of pregnant women to clinical disorders caused by excessive iron [3].

In a comprehensive review presented at the workshop *“Iron Screening and Supplementation in Iron-Replete Pregnant Women and Young Children”* held by the NIH Office of Dietary Supplements in 2016, Dewey & Oaks confirmed the U-shaped association curve reported in previous epidemiologic studies [73,74,75,76] between maternal hemoglobin concentrations or iron status during pregnancy and the risk of adverse birth outcomes, with a higher risk of LBW and PTB in women with both low and high circulating hemoglobin concentrations [77]. For low hemoglobin concentrations, the association is strongest in early pregnancy and appears to wane in the second or third trimesters. For high hemoglobin concentrations, the association with adverse pregnancy outcome appears to remain elevated throughout all three trimesters, although the data are less consistent [77]. The few studies that directly measured iron status show a significant association between high iron status and increased risk of PTB and LBW in all three trimesters. Of note, these studies measured serum ferritin levels, which complicates the interpretation because ferritin is an acute-phase protein that can increase in response to numerous inflammatory or infectious stimuli [78] or could simply reflect inadequate plasma volume expansion in pregnancy. Data from three studies that reported circulating concentrations of soluble Tf receptor, a more reliable measure of iron status, suggest that a high iron status in late pregnancy is associated with a significantly smaller birth weight [77].

In a randomized controlled trial to evaluate the effect of iron supplementation on pregnancy outcomes in iron-replete women, Ziaei et al. reported that women who had higher mean hemoglobin concentrations in the third trimester were significantly more likely to develop hypertensive disorders (2.7% *vs.* 0.8%, *p* < 0.05) and deliver small-for-gestational age (SGA) infants (15.7% *vs*. 10.3%, *p* = 0.035) [79]. Another observational study conducted on non-anemic South Indian pregnant women showed that infants of mothers who took high doses of supplemental iron (41.5 mg/day) had a shorter duration of gestation and higher risk of term LBW (16.8% *vs.* 8.5%; adjusted OR: 1.89; 95% CI: 1.26–2.83) [80]. Taken together, these data suggest that routine iron supplementation in iron-replete women does not translate into improvements in perinatal outcome, but rather appears to be associated with significantly more adverse pregnancy events.

The molecular mechanisms underlying the association between excessive iron intake or high iron status during pregnancy and adverse birth outcomes remain unclear, but several potential mechanisms have been proposed [77]:

- In iron-replete women, prenatal iron supplementation throughout pregnancy may suppress maternal hepcidin production and/or activity [81], leading to continuous absorption of dietary iron, higher hemoglobin concentrations, and increased blood viscosity, ultimately compromising placental blood flow [79].
- Excessive dietary iron intake may lead to a postprandial increase in circulating levels of NTBI, contributing to oxidative stress, lipid peroxidation, and DNA damage in placental cells [82,83].
- Excess iron may impair the maternal systemic response to inflammation and infection leading to adverse birth outcome [84].
- Lastly, excess iron may alter the maternal gut microbiome [85] and/or increase the risk of copper and zinc deficiency [86,87], which in turn may culminate in adverse pregnancy outcomes.

### 3.2. Maternal Iron Status and Preeclampsia

Preeclampsia (gestational hypertension with maternal end-organ damage) complicates 5–7% of all pregnancies [88]. It is a major cause of maternal morbidity and mortality, second only to venous thromboembolic disease in developed countries. A woman dies somewhere in the world every 8 minutes from complications of PE. Moreover, women who survive a PE pregnancy are at significantly increased risk of cardiovascular disease, stroke, and early death remote from delivery [89,90]. Whether this is due to common risk factors or persistent endothelial injury due to PE is not known [88,89,90,91]. Preeclampsia is also a major cause of perinatal morbidity and mortality, due primarily to iatrogenic prematurity. Preeclampsia cannot currently be prevented to any significant degree, although low-dose aspirin prophylaxis is commonly recommended to women at high-risk. The only effective treatment for PE is delivery. It is also a major driver of healthcare costs [92]. A better understanding of the pathogenesis of PE is urgently needed if we are to impact maternal and perinatal outcome.

Preeclampsia is a systemic multisystem disorder specific to pregnancy and the puerperium; it does not occur in men or in women outside of this time period. Moreover, it is a disease specific to humans, although there are scattered reports of PE-like syndromes occurring in non-human great apes, but only those that have human-like deep placental invasion (gorillas and chimpanzees) [93]. Several animal models of gestational proteinuric hypertension have been developed [94,95], but it is not clear that any of these accurately recapitulate the clinical syndrome seen in humans.

Preeclampsia is a disease of the placenta. In the absence of an adequate animal model, the data in support of this assertion are largely indirect. For example, PE is seen more commonly in pregnancies where there are excessive trophoblasts, but no fetal tissue (complete molar pregnancies) [96]. Moreover, if PE develops in patients with an advanced intra-abdominal extrauterine ectopic pregnancy, the disease fails to resolve after delivery because the source of the disease (the placenta) cannot be removed [97]. Preeclampsia can be divided into two broad categories [98,99,100]: late- and early-onset PE. (i) Late-onset PE occurs at or near term and is thought to reflect placental dysfunction due to abnormalities in the maternal vasculature. For this reason, it is also referred to as ‘maternal injury’ preeclampsia. Risk factors include chronic hypertension, renal disease, diabetes with vasculopathy, and collagen vascular disease. (ii) Early-onset PE, also known as ‘placental injury’ PE, classically presents before 34 weeks of gestation. The cause appears to be suboptimal placental perfusion resulting from a primary defect in placental development. Risk factors include hydatidiform mole, placentomegaly, fetal hydrops (so-called “mirror syndrome”), antiphospholipid antibody syndrome, fetal aneuploidy, and confined placental mosaicism.

Early-onset PE is the more serious variant, since it presents earlier in gestation and is more likely to have severe manifestations [88]. Despite decades of research, the etiology of early-onset PE remains elusive. That said, there does appear to be a consistent pathologic hallmark, specifically failure of the extravillous cytotrophoblast cells (EVCTs) to adequately remodel the maternal spiral arteries, leading to shallow endovascular invasion of the placenta [98,99,100,101,102,103,104,105,106]. This localized vascular remodeling is critical for the establishment of the definitive uteroplacental circulation. Preeclampsia is thus a two-stage disease. The blueprint for the disease is laid down early in pregnancy with abnormal implantation and placentation between 8 and 18 weeks of gestation. Later in gestation, as the feto-placental unit outgrows its blood supply, the placenta becomes dysfunctional and releases as yet unidentified factors into the maternal circulation that result in generalized endothelial injury, leading to the clinical manifestations of the maternal syndrome we know as PE [106]. Some investigators have suggested that PE may actually be a three-stage disease, starting with abnormal immunological priming of the endometrium even before the blastocyst arrives, a process mediated, in part, by exposure to sperm antigens and seminal fluid [107,108].

The placenta is developmentally crucial for reproductive success and is the most conspicuous anatomical novelty of placental (eutherian) mammals. However, before it can exert its dual functions as an endocrine organ and an organ capable of facilitating gas and nutrient exchange, enormous changes must take place within the uterus to tolerate the presence of this hemi-allogeneic tissue and to accommodate and support placental development [109]. In placental mammals, the vascularized membranes of the yolk sac and/or allantois fuse with the chorion to form the placenta, which then attaches to the uterine wall. Although nutrient and gas exchange with maternal blood is common to all placentas, the degree of invasiveness into the maternal tissues of the uterus varies from species to species, with the human chorioallantoic placenta being the most invasive of all [109,110]. A clear understanding of implantation and placentation is needed if we are to understand the underlying pathogenesis of shallow endovascular invasion and PE.

Women ovulate 14 days before their next menstrual cycle. In the presence of viable spermatozoa, fertilization occurs within 24–48 h. The resultant zygote develops into a morula and then a blastocyst as it makes its way down the fallopian tube. The blastocyst enters the uterine cavity approximately day 5 postconception, hesitates, and then starts to invade into the endometrial lining starting on day 7 postconception. By day 10 postconception, it is entirely buried and covered by the endometrial lining. This is known as the ‘first wave’ of trophoblast invasion. Over the next few weeks, the embryo is nourished by secretions from the endometrial glands (so-called histiotrophic support) and exists in an environment that is both hypoxic and hypoglycemic. Indeed, exposure to excess glucose or oxygen is toxic to the developing embryo. Diabetic embryopathy (the teratogenic effect resulting from high circulating glucose levels around the time of conception) is a well-known phenomenon [111]. Less well known is the teratogenic effect of high levels of oxygen, thought to be caused by the production of reactive oxygen species (ROS). Hypoxia and the resultant upregulation of hypoxia-inducible factors within the tissues of the uterus are considered important elements of normal implantation and early placental development [112,113]. Indeed, culturing human embryos in 5% oxygen concentration yields higher blastocyst formation rates, more optimal embryos, and more favorable clinical outcomes than culturing embryos with atmospheric (21%) oxygen [114,115]. A recent study showed that human embryos cultured under ultralow oxygen levels (2%) were even more likely to blastulate without arrest compared with the 5% oxygen group (adjusted OR, 2.55; 95% CI, 1.27–5.12) [116].

At around 8-10 weeks of gestation, EVCTs at the site of the early placenta change their adhesion molecule expression and stream out of the placental villi to invade the full thickness of the decidualized endometrium (decidua) and the inner third of the myometrium. These EVCTs target the maternal spiral arteries, attracted in part by the high oxygen tension [117]. Having identified the spiral arteries, the invading EVCTs immediately set about remodeling them in two ways: (i) by replacing the endothelial lining with a pseudo-endothelium of fetal origin, and (ii) by destroying the muscle layer of these vessels. This process—known as the ‘second wave’ of trophoblast invasion—is usually complete by 18 weeks of gestation and is critical for the establishment of the definitive utero-placental circulation. As the pregnancy progress, the 120–140 tiny, tortuous maternal spiral arteries that supply each placenta need to dilate enormously to accommodate the increasing demands of the placenta and fetus. The placenta is a high-volume, low-resistance organ. At term, almost one-fifth of the maternal cardiac output (approximately 800 mL) passes through the placenta every minute. If the EVCTs fail to adequately remodel the maternal spiral arteries from narrow lumen, tortuous vessels with a thick coating of muscle to wide, thin-walled, funnel-shaped vessels, a process that is usually complete by 18 weeks of gestation, the feto-placental unit will rapidly outgrow its blood supply resulting in suboptimal oxygenation and nutrition. The end result will be placental dysfunction and the clinical syndrome in the mother we recognize as PE, with or without fetal growth restriction. In Section 4.3. below, we will propose a mechanism whereby iron overload may lead to ferroptosis, suboptimal placental invasion, and ultimately PE.

## 4. Molecular Basis for Dysregulated Iron Homeostasis in Preeclampsia

### 4.1. Ferroptosis, an Iron-Dependent Mechanism of Programmed Cell Death

**Figure 3:**
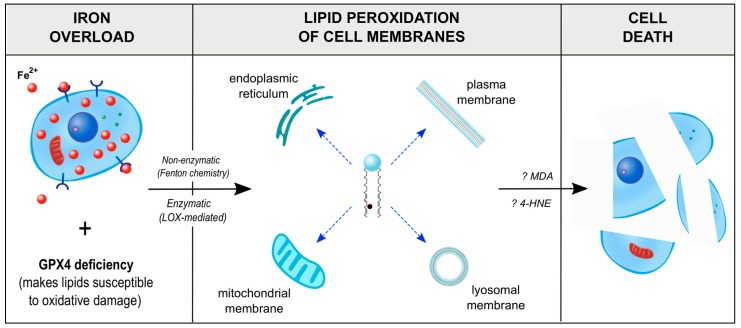
Proposed cellular mechanisms of ferroptosis, which refers to programmed cell death mediated by iron-dependent lipid peroxidation within cell membranes.

The term ‘ferroptosis’ was coined in 2012 to describe a newly-identified mechanism of programmed cell death mediated by iron and distinct from other known forms of programmed cell death, such as apoptosis [118]. A number of publications have since confirmed the existence of ferroptosis and characterized its impact in both health and disease [119,120,121]. Ferroptosis is perhaps best described as programmed cell death mediated by the iron-dependent lipid peroxidation of cell membranes. Stated differently, ferroptosis is cell death caused by lipid peroxidation of cell membranes, which is itself caused by excess iron. The first description of the mechanism of ferroptosis involved the action of erastin, named for eradicator of RAS and small T antigen-expressing cells, a small molecule that inhibits the cystine/glutamate antiporter system and, thereby, limits the intracellular availability of cysteine required for glutathione synthesis. The depletion of glutathione and inactivation of GPX4 (glutathione peroxidase 4) [122], a phospholipid hydroperoxidase that protects cells against lipid peroxidation, results in membrane lipid peroxidation that builds up to toxic levels and kills the cell [123] (Figure 3). Other than dysregulated amino acid (glutathione) metabolism, additional processes that can initiate and execute lethal iron-dependent membrane lipid peroxidation, including abnormalities in metabolic processes involving iron, polyunsaturated fatty acids (PUFAs), phospholipids, NADPH, and coenzyme Q_10_ [121].

The most common lipids for triggering ferroptosis belong to the PUFA-containing membrane phospholipid family [124]. Lipidomic studies suggest that phosphatidylethanolamines containing arachidonic acid (C20:4) or the elongation product, adrenic acid (C22:4), are key phospholipids that undergo oxidation and promote ferroptotic cell death [125,126]. The source of iron involved in membrane lipid peroxidation likely comes from the labile iron pool within cells. Two iron-dependent lipid peroxidation mechanisms are described: non-enzymatic and enzyme-dependent processes. (i) Non-enzymatic free-radical chain reactions involve Fenton chemistry that generates highly toxic hydroxyl and peroxyl radicals. (ii) Enzyme-dependent processes involve iron-containing enzymes such as lipoxygenases [121,124]. Seiler et al. have reported that 12/15-lipoxygenase-mediated lipid peroxidation as a downstream cell death pathway of GPX4 inactivation [127]. Ferroptosis can thus be regulated by factors that directly or indirectly target iron metabolism and lipid peroxidation. For example, ferroptosis can be triggered by exposure to excess iron or agents that increase intracellular ROS (such as NADPH oxidase), and it can be inhibited pharmacologically by iron chelators (deferoxamine), lipophilic antioxidant (ferrostatin), and enzymes that reduce lipid hydroperoxides and cellular iron uptake [120], but not by inhibitors of apoptosis or necroptosis [123].

The mechanism by which lipid peroxidation causes ferroptosis, and precisely where in the cell this occurs, are questions that are under active investigation [124]. Feng & Stockwell suggest that many cellular organelles may be involved, including the plasma membrane (disruption of which could compromise the integrity of the cell), lysosomes (the site of ferritinophagy [38,39]), the endoplasmic reticulum (which contains the largest pool of lipids in cells), and the mitochondrial membrane [124].

Dysregulated ferroptosis has been implicated in multiple disease states, including cancer, neurodegenerative diseases, acute renal failure, drug-induced hepatotoxicity, hepatic and heart disease, T-cell immunity, and in ischemia/reperfusion injury [120,121]. Specifically, it has been reported that ferroptosis is an important mediator of kidney [128] and heart injury [129] in the setting of ischemia/reperfusion. The role of ferroptosis in pregnancy-related disorders has not yet been examined.

### 4.2. Expression of Key Iron Homeostatic and Ferroptosis Regulatory Genes at the Maternal-Fetal Interface—Analysis of a Single-Cell RNA-Seq Dataset

To evaluate the expression of genes involved in iron homeostasis and ferroptosis in cells at the maternal-fetal interface, we analyzed a placental single-cell RNA next-generation sequencing dataset for trophoblasts, dendritic cells, stromal fibroblasts, and decidual cells published in 2017 by Pavlicev et al. [130]. Results are presented in Table 1.

Analysis of the placental single-cell RNA next-generation sequencing dataset [130] with a particular focus on iron homeostasis and ferroptosis genes provided three major insights:

- Ferritin genes, in particular *Ftl*, are highly expressed in almost all cell types, suggesting that intracellular iron storage and prevention of iron toxicity is likely of high importance at the maternal-fetal interface. The *Fpn* gene is highly expressed in trophoblasts and decidual cells relative to endometrial stromal fibroblasts. The hepcidin-FPN feedback loop is known to operate in placental syncytiotrophoblast to control iron trafficking from the placenta to the fetus [14]. Studies have also shown that FPN function varies in different organs. Deletion of the *Fpn* gene in mice is embryonic lethal due to a lack of iron transfer to the embryo [14]. In contrast, conditional *Fpn* gene deletion in intestinal enterocytes causes severe anemia. Similarly, conditional deletion in macrophages or hepatocytes is not lethal and only causes anemia when dietary iron is restricted [41]. Conditional *Fpn* gene deletion in cardiomyocytes causes premature cell death, suggesting that FPN may have a specific role in the heart to protect these cells from iron toxicity [131]. Whether FPN has a similar protective function in decidual cells is not known. Cellular iron homeostasis in decidual cells is likely regulated by IRP2, since the *Irp2* gene is highly expressed in this cell type.
- Decidual cells likely take up iron via both the Tf and NTBI routes, as these cells have high expression levels of the Tf receptor *Tfrc* gene (about 3-fold higher than other cell types), *Dyctb* ferrireductase gene, and *Dmt1* gene, all critical components of the Tf uptake pathway, as well as high expression of the *Zip14* (*SLC39A14*) gene, important for NTBI import. Trophoblast cells, on the other hand, likely import iron as heme via the heme importer protein, FLVCR2 [132], and mitoferrin (*SLC25A37* gene). The iron might be used for heme biosynthesis in the fetal compartment as suggested by the high expression of the *Alas1* (δ-aminolevulinate synthase 1) gene, which codes for a rate-limiting enzyme in the heme biosynthetic pathway.
- Trophoblast cells are likely more vulnerable to ferroptosis than other cell types at the maternal-fetal interface based on the high expression of two genes: (i) *Lpcat3* (lysophosphatidylcholine acyl-transferase 3), whose gene product is involved in the biosynthesis and remodeling of PUFA-phosphatidyl-ethanolamine phospholipids in cell membranes and is a critical mediator of ferroptosis [126,133]; and (ii) *Sat1* (spermidine/spermine N1-acetyltransferase 1), which codes for an important enzyme in polyamine metabolism that also promotes p53-dependent ferroptotic responses [134].

Taken together, these data suggest that different cell lineages at the maternal-fetal interface have different modes of intracellular iron regulation and distinctive sensitivities to ferroptosis. Trophoblast cells are likely more vulnerable to membrane lipid peroxidation and ferroptosis, which may have implications for the development of disorders such as PE (discussed below).

### 4.3. Abnormal Ferroptotic Response to Hypoxia/Reperfusion: A Novel Model for Preeclampsia

**Figure 4:**
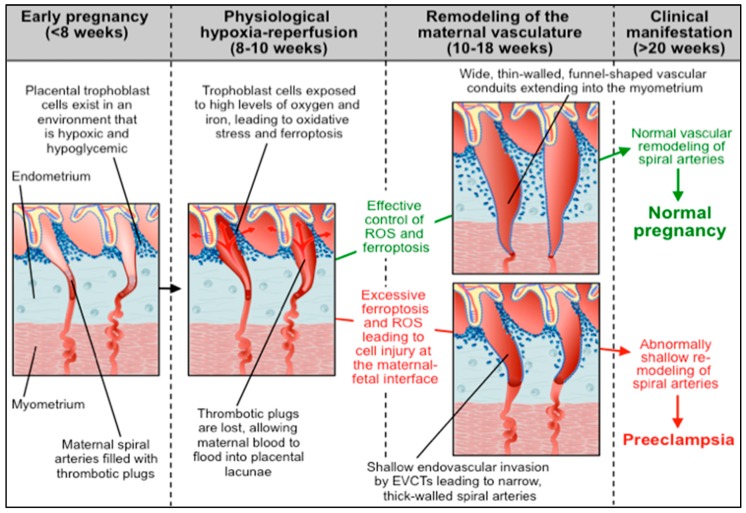
A novel model for preeclampsia: excessive ferroptosis and cellular injury at the maternal-fetal interface in response to the physiologic hypoxia-reperfusion events that occurs in all pregnancies at 8–10 weeks of gestation.

Up until 8–10 weeks of gestation, the maternal spiral arteries remain completely obstructed by congealed endothelial cells and blood clots. During this time, the embryo exists in an environment that is hypoxic and hypoglycemic. At around 10–12 weeks of gestation, the spiral arteries become fully canalized and maternal blood floods into the placental lacunae, exposing the fetal villi for the first time to an environment rich in glucose, oxygen, and iron. Much like the hypoxia/reperfusion event that occurs after surgical correction of an acute vascular occlusion, one would expect this rapid reperfusion to cause massive oxidative stress and tissue injury. We propose that some pregnancies generate an exaggerated response to the acute surge in oxygen and iron caused by the physiologic hypoxia/reperfusion event that occurs in all pregnancies at 8–10 weeks of gestation resulting in cell membrane lipid peroxidation and excessive ferroptosis at the maternal-fetal interface, primarily in trophoblast cells, leading to shallow endovascular invasion of EVCTs and suboptimal remodeling of the maternal spiral arteries, the pathologic hallmarks of PE (Figure 4).

There is substantial, albeit indirect, evidence to support this hypothesis, summarized below:

- Numerous studies investigating the association between iron status and hypertensive disorders of pregnancy have noted that levels of circulating iron and ferritin, as well as the level of Tf saturation, are significantly higher in PE patients as compared to normotensive controls [135,136,137]. Whether or not the increased serum iron in PE is due to low circulating hepcidin levels is unclear. Studies by Koenig et al. [81] and by Brunacci et al. [138] suggest that high serum iron and Tf saturation in PE women correlate with lower hepcidin levels. However, a study by Duvan et al. found no significant correlation between prohepcidin concentrations and serum iron, ferritin, or Tf levels in women with PE [139].
- Several studies have shown that serum levels of malondialdehyde (MDA), the most mutagenic product of lipid peroxidation [140] and an oxidation product that likely contributes to ferroptosis [124], are significantly elevated in patients with PE and eclampsia [141,142,143].
- A metabolomic analysis of placental mitochondria has identified that severe PE patients have significantly higher PUFA levels and other mitochondrial abnormalities relative to normotensive controls [144].
- Proteomic analysis has shown that patients with severe PE have significantly higher concentrations of free α- and β-hemoglobin in their cerebrospinal fluid (CSF) as compared to women with non-severe PE or normotensive controls [145]. Similarly, van den Berg et al. reported that CSF levels of the heme-binding protein, AMBP (α-1-microglobulin/bikunin precursor), are markedly elevated in women with severe PE relative to normotensive controls [146]. Taken together, these data suggest that patients with PE may have difficulty disposing of free hemoglobin and associated proteins.
- In many cases of PE, lipid-filled ‘foam cells’ (macrophages containing low density lipoproteins) accumulate in the walls of the spiral arteries, reminiscent of the early stages of atherosclerosis [147]. Although this histologic aberration was observed decades ago, the mechanisms that contribute to this acute atherosis are largely unknown. Ferroptosis with lipid peroxidation could be a contributing factor.

The role of mitochondrial dysfunction in this model deserves further attention. Our analysis of the single-cell RNA sequencing dataset in Section 4.2. suggests that trophoblast cells are more vulnerable to ferroptosis than endometrial or decidual cells. Since most intracellular iron is utilized in mitochondria for heme and Fe-S cluster biosynthesis, and since mitochondria play a significant role in ferroptosis [148], we speculate that the membranes of trophoblast mitochondria might exhibit significant lipid peroxidation and induce ferroptosis. Indeed, studies have shown that hypoxia/reoxygenation of healthy tissues changes placental mitochondrial function in a manner similar to that seen in PE pregnancies [149]. Mitochondria-derived from gestational diseases, including PE, exhibit changes in number, size, and morphology [150], are functionally ‘lethargic’ [144], and show evidence of increased oxidative stress [151,152]. Interestingly, placentas from PE pregnancies that reach term show adaptations in mitochondrial function that may represent an effort to ameliorate the deficiencies seen in preterm PE pregnancies [149]. Cytotrophoblast differentiation into syncytiotrophoblast involves changes in mitochondrial appearance and function [153]. Mitochondria from multinucleate syncytiotrophoblast are structurally different from those isolated from cytotrophoblasts and contain large amounts of cytochrome p450 heme-containing enzymes and heat-shock proteins for steroidogenesis and superoxide generation [154]. These syncytiotrophoblast mitochondria have a low oxygen metabolism, increased ROS production, and are less likely to undergo apoptosis if perturbed [154]. However, these same changes make them far more vulnerable to initiating ferroptosis when confronted with disturbances in iron homeostasis.

Mitochondria have an obligate need for iron. As such, a mechanism exists within cells to coordinate cytosolic and mitochondrial iron trafficking such that a disruption in mitochondrial homeostasis (e.g., inhibition of heme or Fe-S cluster biogenesis) will trigger a compensatory response leading to increased iron uptake and transport into the mitochondria. If dysregulated, this can lead to iron overload in mitochondria and iron deficiency in the cytosol (see *Fxn* knockout murine model and other studies in Section 2.2.) [58,59,63]. It would be important, therefore, to examine more closely both mitochondrial and cytosolic parameters to understand the value of iron homeostatic perturbation and ferroptosis in the pathogenesis of PE.

Alternative models for the role of ischemia/reperfusion and iron excess in pregnancy-related complications have been proposed. For example, repetitive ischemia/reperfusion in early pregnancy has been suggested as a cause for adverse pregnancy events, including miscarriage and PE, although the proposed mechanism was one of chronic, rather than acute, oxidative stress [155]. Alahari et al. suggested that PE results from chronic hypoxia at the maternal-fetal interface. They described a mechanism that includes hypoxia- and iron-mediated downregulation of the histone demethylase, JMJD6 (Jumonji domain-containing protein 6), and subsequent epigenetic dysregulation of the *Vhl* (von Hippel Lindau) tumor suppressor gene, which is important for oxygen sensing, HIF1α signaling, and angiogenesis [156]. Whether lipid peroxidation and ferroptosis are involved in these models has not been investigated.

## 5. Conclusions

For clinical care providers, oral iron supplementation for the treatment of iron-deficiency anemia is considered effective, inexpensive, and safe in pregnancy. In non-anemic pregnant patients, however, routine iron supplementation is likely unnecessary and, if recommended, should probably be limited to less than 60 mg daily or every other day. Alternate day administration is associated with fewer gastrointestinal side-effects. Many epidemiologic studies have shown that excessive iron intake and/or high iron status can be detrimental to pregnancy and is associated with the development of a number of reproductive disorders. We posit that this is due to an underlying ferroptopathy characterized by intracellular iron excess leading to ferroptosis, a process of programmed cell death mediated by iron-dependent lipid peroxidation of cell membranes. Ferroptosis has been shown to play an important role in sterile inflammatory conditions such as hypoxia/reperfusion injury. By way of illustration, we propose that some pregnancies generate an exaggerated response to the acute surge in oxygen and iron caused by the physiologic hypoxia/reperfusion event that occurs in all pregnancies at 8–10 weeks of gestation. This surge of oxygen and iron results in excessive membrane lipid peroxidation and ferroptosis at the maternal-fetal interface, primarily in trophoblast cells, leading to shallow endovascular invasion of EVCTs and suboptimal remodeling of the maternal spiral arteries, the pathologic hallmarks of PE. A better understanding of the role of ferroptosis in pregnancy-related disorders, including PE, will provide new opportunities for diagnosis and therapeutic interventions.

## Figures and Tables

**Table 1:** Expression of iron homeostatic and ferroptotic genes in different cell types at the maternal-fetal interface at term *.

**Highly expressed genes (within the top 1000 ranked highly expressed genes in any cell type)**								
**GENE**	**CYT1**	**CYT2**	**CYT3**	**DC**	**EVCT**	**SYN**	**ESF**	**DEC**
***Ftl***	2807 (54)	5427 (24)	4936 (29)	26753 (2)	15921 (3)	19339 (4)	4849 (12)	12331 (3)
***Fth1***	493 (318)	1224 (128)	722 (227)	5411 (29)	1093 (163)	1348 (92)	3416 (19)	7596 (6)
***Slc40a1 (Fpn)***	580 (289)	326 (467)	294 (503)	22 (2332)	2 (7287)	51 (1627)	1 (10,661)	317 (611)
***Tfrc (Tfr1)***	227 (633)	217 (666)	203 (717)	191 (731)	22 (3187)	26 (2860)	236 (800)	591 (282)
**Genes mostly expressed in endometrial stromal fibroblasts and decidual cells**								
**GENE**	**CYT1**	**CYT2**	**CYT3**	**DC**	**EVCT**	**SYN**	**ESF**	**DEC**
***Cybrd1 (Dyctb)***	0	1	0	0	0	45	334	334
***Flvcr1***	1	0	2	0	0	1	12	7
***Fxn***	0	4	2	3	0	0	21	12
***Ireb2 (Irp2)***	1	6	6	0	9	18	47	68
***Pcbp4***	0	0	0	0	0	4	39	18
***Slc7a11***	0	0	0	0	0	0	76	66
***Zip14 (Slc9a14)***	0	3	0	0	0	2	205	280
**Genes mostly expressed in trophoblasts**								
**GENE**	**CYT1**	**CYT2**	**CYT3**	**DC**	**EVCT**	**SYN**	**ESF**	**DEC**
***Alas1***	150	348	472	12	205	41	14	18
***Cp***	37	5	1	3	0	2	0	0
***Flvcr2***	8	12	24	0	11	4	1	1
***Lpcat3***	46	86	77	0	13	109	13	26
***Sat1***	10937	3510	4303	6383	9724	548	97	629
***Mfrn (Slc25a37)***	12	88	122	0	493	19	57	36
**Genes expressed in both trophoblasts and stromal fibroblasts and decidual cells**								
**GENE**	**CYT1**	**CYT2**	**CYT3**	**DC**	**EVCT**	**SYN**	**ESF**	**DEC**
***Abcb10***	8	7	2	1	6	16	30	14
***Aco1 (Irp1)***	10	43	30	53	14	8	13	24
***Fech***	7	22	28	5	10	2	55	60
***Hmox1***	111	249	82	2	53	25	114	113
***Hmox2***	2	8	66	0	15	8	56	32
***Pcbp1***	175	314	265	60	175	178	192	152
***Pcbp2***	138	152	174	225	144	66	179	153
***Dmt1 (Slc11a2)***	3	30	5	26	2	25	33	72
***Mfrn2 (Slc25a28)***	27	34	53	0	10	4	40	25
***Zip8 (Slc39a8)***	0	4	7	25	48	3	22	14
***Hrg1 (Slc48a1)***	35	184	305	0	31	3	61	173
***Steap3***	39	19	15	17	0	1	9	3
**Downregulated genes**								
**GENE**	**CYT1**	**CYT2**	**CYT3**	**DC**	**EVCT**	**SYN**	**ESF**	**DEC**
***Hamp (Hepcidin)***	0	0	0	0	0	0	0	0
***Hpx***	0	0	2	0	0	0	0	0

* The data were derived from Table S1 of the reference manuscript [130]. Numbers represent normalized expression in transcripts per million (TPM) relative to the total of coding genes. For the highly expressed gene category, the ranking of the gene relative to all genes identified within each cell type is shown in parentheses. The study found that there were three types of placental (villous) cytotrophoblasts (CYT), designated CYT1, CYT2, and CYT3. Abbreviations: DC, dendritic cells; DEC, decidual cells; ESF, endometrial stromal fibroblasts; EVCT, extravillous cytotrophoblasts; SYN, syncytiotrophoblast.

## Abbreviations

ABCB10	ATP Binding Cassette super-family B member 10
Alas1	δ-aminolevulinate synthase 1
AMBP	α-1-Microglobulin/Bikunin Precursor
Apo-Tf	Apo-Transferrin
CD163	Cluster of Differentiation 163
CI	Confidence Interval
CP	Ceruloplasmin
CSF	Cerebrospinal Fluid
CYT	Cytotrophoblast
DC	Dendritic Cell
DCYTB	Duodenal Cytochrome B
DEC	Decidual Cell
DMT1	Divalent Metal Transporter 1
DNA	Deoxyribonucleic Acid
ESF	Endometrial Stromal Fibroblast
EVCT	Extravillous Cytotrophoblast
FBXL5	F-Box and Leucine rich repeat protein 5
Fe^2+^	Ferrous Iron
Fe^3+^	Ferric Iron
FECH	Ferrochelatase
Fe-S cluster	Iron-Sulfur Cluster
FLVCR1	Feline Leukemia Virus subgroup C Receptor-related protein 1
FPN	Ferroportin
FTH1	Ferritin Heavy chain 1
FTL	Ferritin Light chain
FTMT	Mitoferritin
FXN	Frataxin
GPX4	Glutathione Peroxidase 4
HEPH	Hephaestin
HIF1α	Hypoxia-Inducible Factor 1 alpha
HIF2α	Hypoxia-Inducible Factor 2 alpha
Holo-Tf	Holo-Transferrin
HMOX1	Heme Oxygenase 1
HMOX2	Heme Oxygenase 2
HRG1	Heme-Responsive Gene-1
IRE	Iron-Responsive Element
IRP1	Iron Regulatory Protein 1
IRP2	Iron Regulatory Protein 2
JMJD6	Jumonji Domain-containing protein 6
LBW	Low Birthweight
LPCAT	Lysophosphatidylcholine Acyl- Transferase 3
MDA	Malondialdehyde
MFRN1	Mitoferrin 1
MFRN2	Mitoferrin 2
NAPDH	Nicotinamide Adenine Dinucleotide Phosphate Hydrogen
NCOA4	Nuclear receptor Coactivator 4
NIH	National Institute of Health
NTBI	Non-Transferrin Bound Iron
OR	Odds Ratio
PCBP1	Poly-(rC)-Binding Protein 1
PCBP2	Poly-(rC)-Binding Protein 2
PE	Preeclampsia
PPIX	Protoporphyrin IX
PTB	Preterm Birth
PUFA	Polyunsaturated Fatty Acid
RES	Reticuloendothelial System
RNA	Ribonucleic Acid
ROS	Reactive Oxygen Species
SAT1	Spermidine/spermine N1-Acetyltransferase 1
SGA	Small-for-Gestational Age
STEAP3	Six-Transmembrane Epithelial Antigen of Prostate 3
SYN	Syncytiotrophoblast
Tf	Transferrin
TfR1	Transferrin Receptor 1
Tfrc	Transferrin Receptor C
TPM	Transcripts per Million
VHL	Von Hippel Lindau tumor suppressor gene
WHO	World Health Organization
ZIP8	Zrt- and Irt-like Protein 8
ZIP14	Zrt- and Irt-like Protein 14
